# GIS-based spatial analysis for lightning scenario in Bangladesh

**DOI:** 10.1016/j.heliyon.2024.e28708

**Published:** 2024-03-28

**Authors:** Ferdous Ahmed, Sakib Hasan, I.M. Mahbubul, Muhammad Abul Kalam Mallik, M. Nafiz Hossen

**Affiliations:** a Department of Environmental Science, International University of Business Agriculture and Technology, 4 Embankment Drive Road, Sector-10, Uttara, Dhaka, Bangladesh; b Department of Civil Engineering, International University of Business Agriculture and Technology, 4 Embankment Drive Road, Sector-10, Uttara, Dhaka, Bangladesh; c Institute of Energy Engineering, Dhaka University of Engineering & Technology, Gazipur, Bangladesh; d Meteorologist, Storm Warning Centre, Bangladesh Meteorological Department, Dhaka, Bangladesh

**Keywords:** Lightning scenario, Lightning fatalities, Geographic Information system (GIS), Bangladesh

## Abstract

Bangladesh has witnessed alarmingly rising lightning frequency, particularly during pre-monsoon and monsoon seasons. This has resulted in significant annual death tolls from lightning strikes over the past decade. Recognizing this crisis, the country officially declared lightning casualties a natural disaster in 2016. This study delves deeper into the landscape of lightning fatalities and causalities in Bangladesh. Utilizing secondary data sources, this research introduces a unique approach by integrating Bangladesh Meteorological Department (BMD) data and NASA's Lightning Imaging Sensor (LIS) data from the International Space Station's (ISS) Near-real Time (NRT) mission. This combined dataset allows for a more comprehensive analysis. Furthermore, Geographic Information Systems (GIS) was employed to analyze spatial distributions and generate maps. The Inverse Distance Weighted (IDW) interpolation tool was used to create detailed spatial distribution maps of lightning fatalities, thunderstorm days (TSDs), and lightning flash frequency (LFF) across Bangladesh. The analysis revealed that farmers and fishermen were the most vulnerable populations, with the northeastern regions experiencing the highest impact. Sylhet division emerged as the area with the most fatalities, highlighting the northeastern zone's susceptibility. The study also identified monsoons as the period with the highest occurrences of lightning deaths and injuries. By combining innovative data integration and spatial analysis, this study offers valuable insights into the alarming trend of lightning fatalities in Bangladesh. These findings can inform targeted prevention strategies and interventions to safeguard vulnerable populations and communities.

## Highlights

• Farmers and fishermen were identified as the most vulnerable groups to lightning strikes.
• The northeastern region of Bangladesh exhibited the highest susceptibility to lightning fatalities.
• Sunamganj leads to significant lightning fatalities followed by Habiganj and Naogaon whereas Pirojpur recorded lowest.
• Geographic location, weather, and climatic factors are attributed to the increased vulnerability of the northeastern region.

## 1 Introduction

Bangladesh, a country highly susceptible to various natural disasters like floods and cyclones, often faces the lesser-known threat of lightning strikes [[1], [2], [3], [4], [5], [6], [7]]. While floods and cyclones cause significant destruction, lightning often goes unnoticed until recent years [8], where its devastating impact and death toll have garnered attention [[9], [10], [11], [12]]. The horrific deaths of 89 people in just two days (May 12–13, 2016) prompted the government to declare lightning a natural disaster and begin data collection on its casualties [13,14]. Over the past five years, annual lightning fatalities have risen to a staggering 300–400 people, with injuries increasing concurrently [15,16]. Bangladesh's lightning fatality rate of 0.9 per million people surpasses even high-income nations [17], making it one of the countries with the highest mortality rates from this natural disaster [18]. Alternatively, it exhibits an intermediate annual mortality rate ranging from 0.5 to 5.0 per million populations [19]. During the period spanning from 1990 to 2016, there were approximately 5468 incidents of injuries and fatalities attributable to lightning, with 3086 cases leading to death and 2382 causing injuries [13]. Notably, recent years have witnessed a worrying increase in both the frequency and deadliness of lightning strikes [20]. Understanding the environmental factors linked to lightning occurrence is crucial to effectively managing and reducing this weather-induced threat in Bangladesh [6,13].

Between 2010 and 2016, over 1476 people in Bangladesh tragically lost their lives to lightning strikes [21]. Unlike thunder, which often accompanies heavy rain and strong winds, lightning strikes with immense speed and generates intense heat, making it far more dangerous and destructive [21]. National Geographic reports that Bangladesh experiences peak lightning activity in May, coinciding with high temperatures and afternoons [13,22]. Since 1981, the Bangladesh Meteorological Department has recorded a steady increase in lightning frequency, attributing it to climate change [23]. They further noted that lightning strikes primarily occur during the pre-monsoon period (March–May), with climate change intensifying their severity each year [5,23]. This is further supported by a climatic analysis, which revealed that approximately 62% of lightning strikes in Bangladesh occur during the pre-monsoon season [6]. In May 2017 alone, Bangladesh witnessed a staggering 59 lightning-related fatalities [5]. Tragically, most deaths happen between the early morning and evening hours, with 93% occurring in rural areas [13]. This highlights the disproportionate impact of lightning strikes on rural communities in Bangladesh, which has seen a sharp rise in casualties in recent years.

According to Department of Disaster Management (DDM) data, a staggering 828 individuals lost their lives to lightning strikes between 2010 and 2016 [16]. In Bangladesh, farmers, fishermen, and livestock ranchers face an acute risk due to their occupations, often working outdoors in exposed environments [7,24]. This vulnerability extends to subsistence farmers, residents in lightning-prone areas, and individuals working in structures lacking proper protection [24]. Global climate change is evident in shifting rainfall and temperature patterns [[25], [26], [27]]. Research suggests a concerning rise in fatalities from lightning, with an estimated 1800 deaths recorded over the past eight years [5]. This figure represents the highest documented death toll from lightning strikes. Further data from the Indian Weather Office and Japan Aerospace Exploration Agency revealed that Bangladesh endures an average of 2400 lightning strikes annually. These stark statistics paint a grim picture, highlighting the urgent need for effective mitigation strategies and increased awareness to protect vulnerable populations.

Climate change is strongly implicated in the increase in lightning strikes in Bangladesh. This change manifests in various natural hazards, including oceanic storms, cyclones, storm surges, sea level rise, altered rainfall patterns, and extreme temperatures. Given the undeniable impact of climate change on these phenomena, it is logical to assume a strong connection between it and lightning activity in Bangladesh and globally. Studies have shown that Bangladesh experiences a high frequency of lightning and thunder due to summer and monsoon season heat, particularly in the northeastern regions with large water bodies like the Haor area^1^ [27]. Generally, April sees the highest temperature increases, triggering water vaporization, leading to rain, cloud formation, and ultimately, lightning. Research suggests that Bangladesh is a hotspot for lightning in the Indian subcontinent, with an average of 40 strikes per square kilometer during the pre-monsoon season (March-May) [18]. Statistics from the Foundation for Disaster Forum revealed a concerning trend: the death toll from lightning strikes rose from 179 in 2011 to 301 in 2012, then fluctuated to 285 in 2013, 210 in 2014, 186 in 2015, 245 in 2016, and reached 205 in 2017 [28].

The alarming rise in lightning fatalities during specific periods each year poses a significant threat to both the government and communities in Bangladesh. This emphasizes the imperative to thoroughly grasp the trajectory of this peril and pinpoint vulnerable environments for proactive mitigation measures. Geographic Information Systems (GIS) technology offers valuable tools for predicting the spatial susceptibility to lightning disasters. Researchers have successfully utilized the Inverse Distance Weighted (IDW) geostatistical interpolation tool within GIS to analyze spatial patterns, variability, and susceptibility of various disaster variables and environmental components [29,30]. This study presents a novel approach by utilizing the IDW technique in GIS to create four crucial maps for Bangladesh: lightning death tolls map, fatality rates vulnerability map, spatial vulnerability of thunderstorm days (TSDs), and spatial vulnerability of lightning flash frequencies (LFFs) in seasonally. Importantly, this study breaks new ground by integrating data from the BMD with NASA's Lightning Imaging Sensor (LIS) datasets obtained through the International Space Station's (ISS) Near-real Time (NRT) mission. This novel approach enables a comprehensive analysis of lightning fatalities and rates across various districts in Bangladesh.

To the best of our knowledge, no previous study has combined the IDW technique, TSDs and LFF data from BMD, and data from ISS LIS NRT to examine lightning hazards. While prior studies have explored the spatial distribution of lightning fatalities and utilized different satellite datasets for lightning flashes, they have not addressed the spatial distribution of TSDs and LFFs based on BMD observation data. This research effectively bridges this gap by offering a more comprehensive understanding of lightning hazards in Bangladesh. By applying GIS-based spatial analysis, this research revealed that the Sylhet division experiences the highest number of fatalities, highlighting the Northeastern zone's vulnerability to lightning strikes. Furthermore, the study identified monsoons as the season with the most lightning-related mortalities and injuries, reiterating the urgency of developing targeted mitigation strategies.

## 2 Materials and methods

### 2.1 Study area

**Figure-1:**
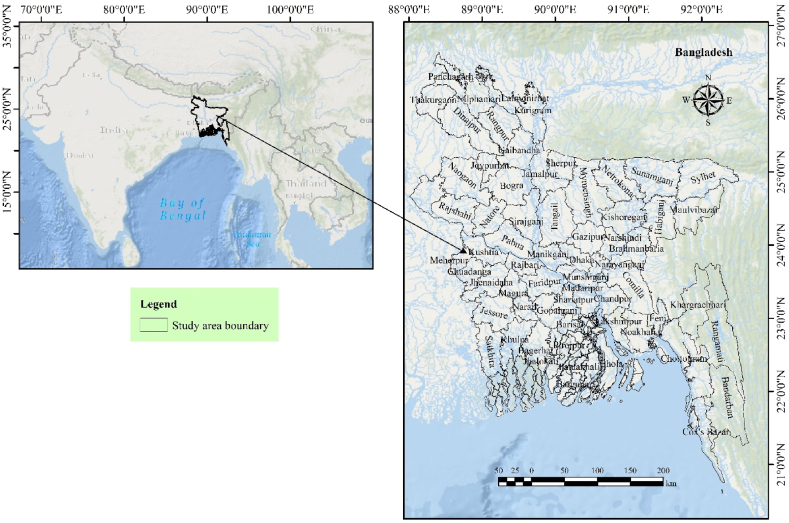
Location of the study area.

Bangladesh, a densely populated nation with approximately 165 million inhabitants according to the 2022 Population and Housing Census [31], sprawls across an area of 147,570 square kilometers between latitudes 20°34′ and 26°38′ North and longitudes 88°01′ to 92°41′ East. The tropical monsoon climate features four distinct seasons: pre-monsoon (March–May), monsoon (June–September), post-monsoon (October–November), and winter (December-February) [23]. Dominated by low-lying land with most areas below 10 m above mean sea level, Bangladesh is intricately woven with the Brahmaputra, Ganges (Padma), Meghna River system, and the Bay of Bengal to the south [32]. This subtropical monsoon country experiences an average annual rainfall ranging from 1110 to 5690 mm, resulting in a humid subtropical climate with temperatures varying between 21 °C and 34 °C throughout the year [33]. This study investigates lightning-related fatalities and injuries across 64 Bangladeshi districts (Fig. 1).

### 2.2 Analytical methods

**Figure-2:**
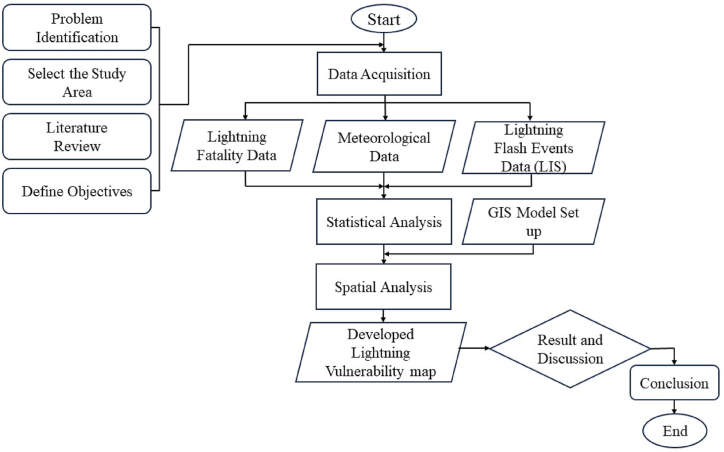
Methodological workflow of the study.

The methodological workflow of this study, illustrated in Fig. 2, follows a sequential approach: (i) this research began with the identification of the lightning strike issue in Bangladesh. After that, (ii) conducted a comprehensive literature review to highlight existing research gaps, followed by (iii) defining precise research objectives. Subsequently, (iv) acquired lightning fatality data from various sources, including research papers and government and non-governmental organizations, (v) collected lightning flash frequency and thunderstorm days' data from the BMD, and (vi) alongside lightning flash events data from the LIS ISS. Then, (vii) applied statistical analysis. The process proceeds with (viii) spatial analysis by employing GIS, and (ix) developed lightning vulnerability map. Finally, (x) rigorously examined the study's findings, and formulated conclusive insights and recommendations based on the results.

### 2.3 Lightning casualties data

Access to comprehensive lightning-related data in Bangladesh is limited. Hence, this study focuses on eight years from 2015 to 2022. Lightning fatality data spanning from 2015 to 2018 was sourced from a relevant research paper [23], while data for the years 2019–2022, including both death and injury statistics, were gathered from yearly and monthly disaster reports published by the Network for Information Response and Preparedness Activities on Disaster (NIRAPAD) [34] and the Bangladesh Department of Disaster Management (DDM) [16]. Lightning flash frequency and average thunderstorm days’ data were obtained from the BMD [35], while Bangladesh and district-wise map shape files were provided by the Bangladesh Agricultural Research Council (BARC) [36].

### 2.4 Lightning flash frequency, thunderstorms days, and LIS data

This study utilizes LFF and TSD data from 35 weather stations across Bangladesh, collected by the BMD between 2015 and 2020. According to the BMD, the pre-monsoon season (March–May) sees the highest concentration of thunderstorms (38%), often associated with the most destructive lightning strikes and casualties. Monsoon season (June–September) experiences 51% of thunderstorms, followed by post-monsoon (October–November) at 8% and winter (December–February) at 3% respectively. However, BMD utilizes a standardized coding practice recommended by the World Meteorological Organization (WMO) Region II to record TSDs and LFF data. These codes include but are not limited to Code 13 (lightning visible, no thunder heard), Code 17 (thunderstorm, but no precipitation at the time of observation), Code 29 (thunderstorm during the preceding hour but not at the time of observation), Code 91 (slight rain at the time of observation), Code 92 (moderate or heavy rain at the time of observation), Code 93 (same as 91, with snow mixed or hill), Code 94 (same as 93 with hail; thunderstorm at the time of observation). Thunderstorm at time of observation; Code 95 (thunderstorm, slight or moderate, without hail), Code 96 (thunderstorm, slight or moderate, with hail), Code 97 (thunderstorm, heavy, without hail but with rain and snow), Code 98 (thunderstorm combined with dust storm or sandstorm) and Code 99 (thunderstorm, heavy, with hail) [35,37]. These codes allow for comprehensive recording of TSD and LFF data from land weather stations across Bangladesh.

Instrumental data was sourced from the LIS aboard the ISS as part of the NRT mission. This mission provides a global dataset of lightning activity, collected by the LIS instrument onboard the ISS. This instrument boasts high detection efficiency, monitoring radiant energy, logging the precise timing, and pinpointing the location of lightning events, even at night. Notably, NRT data is available in low-latency, 2-min intervals. The spatial resolution of this data ranges from 4 to 8 km, with data collection starting in 2017. The LIS instrument employs a wide Field-of-View (FOV) optics lens with a narrow-band filter centered at 777 nm, combined with a high-speed charge-coupled device (CCD) detection array. This configuration optimizes lightning detection and location with a storm-scale resolution of 4 km at the center (nadir) and 8 km at the edge (limb). For this study, we analyzed ISS LIS NRT data from 2017 to 2022 [38]. A summary of the datasets used, including the ones mentioned above, is provided in Table 1.

**Table-1:** ISS LIS NRT Datasets Applied in this Study.

Characteristic	Description
Platform	International Space Station (ISS)
Instrument	Lightning Imaging Sensor (LIS)
Projection	Centroid
Spatial Extent	N: 55.0, S: 55.0, E: 180.0, W: 180.0
Spatial Resolution	4–8 km
Temporal Coverage	March 1, 2017 – ongoing
Temporal Resolution	Near-real Time (NRT) 2 min
Sampling Frequency	Every 2 ms over ∼90 s
Parameter	Lightning, Lightning Density

### 2.5 Data processing and GIS modeling

This case study examines the current state of lightning strikes in Bangladesh, particularly their spatial distribution. GIS plays a crucial role in analyzing data and generating spatial distribution maps. This study utilized the IDW interpolation tool within GIS to reveal the spatial patterns of lightning activity in Bangladesh. Data used includes the average population density per million obtained from the 2022 Bangladesh Housing and Population Census by the Bangladesh Bureau of Statistics (BBS) [31]. Leveraging this data, the study estimated the fatality and injury rate per million population per year and density per square area. GIS tools were then employed to create spatial distribution maps. To calculate the rate of lightning fatalities and injuries per million people per year, the number of recorded deaths and injuries was multiplied by 1,000,000 and then divided by the total population number and study period [39]. Data on lightning casualties and injuries spanned from 2015 to 2022, with the last eight years analyzed using GIS. The specific equations used to analyze the rate of lightning deaths and injuries (Equations (1), (2))) are derived from previous research [5,40].

(1) $$R=\frac{\left[\left(\frac{\sum N}{P}\right)\times 1000000\right]}{n}$$

Here,

R = Lightning casualty/injury rate (per million per year)

∑N = Total number of lightning-related casualties/injuries during the study period (2015–2022)

P = Average population in every district

n = Study period (2015–2022) (Study period is 8 years)

(2) $$D=\frac{\sum N}{A}$$

Here,

D = Lightning casualty/injury density (per sq. km area)

∑N = Total number of lightning-related casualties/injuries during the study period (2015–2022)

A = Area of every district (sq. km)

## 3 Results and discussion

### 3.1 Lightning fatalities and spatial distribution in Bangladesh

Lightning strikes are a growing natural catastrophe in Bangladesh, causing significant fatalities and injuries. Since 2016, this phenomenon has become a major concern, with a worrying trend of casualties. In the past eight years (2015–2022), a total of 2142 deaths and 538 injuries have been recorded due to lightning strikes across 64 districts in Bangladesh (as shown in Table 2).

**Table-2:** **Lightning-related fatalities and injuries in 64 bangladeshi districts (2015**–**2022)**.

District Name	Total Death 2015–2022	Rank	Total Injured 2015–2022	Rank	Average Population (m)	Area (Km^2^)	Time (Years)	Fatality rate		Fatality density		Injury rate		Injury density	
								Rate	Rank	Density	Rank	Rate	Rank	Density	Rank
Barguna	18	44	2	48	1.01	1831	8	2.23	21	0.010	47	0.25	41	0.001	47
Barisal	16	48	2	48	2.57	2785	8	0.78	55	0.006	59	0.10	51	0.001	50
Bhola	20	41	6	31	1.93	3403	8	1.29	44	0.006	58	0.39	28	0.002	44
Jhalokati	12	58	2	48	0.66	749	8	2.27	20	0.016	29	0.38	30	0.003	34
Patuakhali	19	42	0	57	1.73	3221	8	1.38	42	0.006	57	0.00	58	0.000	58
Pirojpur	5	64	0	57	1.20	1308	8	0.52	60	0.004	61	0.00	58	0.000	58
Bandarban	6	61	3	41	0.48	4479	8	1.56	34	0.001	64	0.78	13	0.001	53
Brahmanbaria	37	19	1	51	3.31	1927	8	1.40	40	0.019	19	0.04	54	0.001	55
Chandpur	30	28	8	23	2.64	1704	8	1.42	39	0.018	23	0.38	29	0.005	23
Chottogram	41	14	3	41	9.17	5283	8	0.56	59	0.008	52	0.04	53	0.001	54
Cumilla	39	16	18	9	6.21	3085	8	0.78	54	0.013	40	0.36	31	0.006	16
Cox's Bazar	34	24	12	16	2.82	2492	8	1.51	36	0.014	36	0.53	25	0.005	21
Feni	13	54	4	37	1.65	928	8	0.99	52	0.014	35	0.30	36	0.004	25
Khagrachhari	23	38	8	23	0.71	2700	8	4.03	4	0.009	51	1.40	5	0.003	32
Lakshmipur	13	54	3	41	1.94	1456	8	0.84	53	0.009	50	0.19	44	0.002	41
Noakhali	37	19	8	23	3.63	3601	8	1.28	45	0.010	44	0.28	39	0.002	39
Rangamati	13	54	0	57	0.65	6116	8	2.51	15	0.002	63	0.00	58	0.000	58
Dhaka	15	51	1	51	14.73	1464	8	0.13	64	0.010	46	0.01	57	0.001	51
Faridpur	38	18	0	57	2.16	2073	8	2.20	22	0.018	21	0.00	58	0.000	58
Gazipur	21	39	8	23	5.26	1800	8	0.50	62	0.012	41	0.19	45	0.004	24
Gopalganj	6	61	0	57	1.30	1490	8	0.58	58	0.004	60	0.00	58	0.000	58
Kishoreganj	78	4	6	31	3.27	2689	8	2.98	8	0.029	5	0.23	42	0.002	38
Madaripur	13	54	0	57	1.29	1145	8	1.26	46	0.011	42	0.00	58	0.000	58
Manikganj	37	19	21	6	1.56	1379	8	2.97	9	0.027	7	1.68	3	0.015	2
Munshiganj	6	61	4	37	1.63	955	8	0.46	63	0.006	54	0.31	34	0.004	26
Narayanganj	16	48	1	51	3.91	700	8	0.51	61	0.023	12	0.03	55	0.001	46
Narshindi	34	24	3	41	2.58	1141	8	1.64	32	0.030	4	0.15	47	0.003	35
Rajbari	11	59	1	51	1.19	1119	8	1.16	49	0.010	48	0.11	50	0.001	49
Shariatpur	24	37	0	57	1.29	1182	8	2.32	17	0.020	16	0.00	58	0.000	58
Tangail	35	23	1	51	4.04	3414	8	1.08	51	0.010	45	0.03	56	0.000	57
Bagerhat	9	60	8	23	1.61	3959	8	0.70	57	0.002	62	0.62	21	0.002	42
Chuadanga	25	36	3	41	1.23	1177	8	2.53	14	0.021	15	0.30	35	0.003	36
Jessore	34	24	5	35	3.08	2567	8	1.38	41	0.013	39	0.20	43	0.002	43
Jhenaidah	18	44	2	48	2.01	1961	8	1.12	50	0.009	49	0.12	49	0.001	48
Khulna	26	32	3	41	2.61	4394	8	1.24	48	0.006	56	0.14	48	0.001	52
Kushtia	26	32	9	21	2.15	1601	8	1.51	35	0.016	28	0.52	26	0.006	17
Magura	15	51	4	37	1.03	1049	8	1.81	29	0.014	33	0.48	27	0.004	29
Meherpur	18	44	6	31	0.71	716	8	3.19	7	0.025	9	1.06	10	0.008	7
Narail	17	47	4	37	0.79	990	8	2.69	12	0.017	25	0.63	20	0.004	27
Satkhira	26	32	37	1	2.20	3858	8	1.48	37	0.007	53	2.11	2	0.010	5
Jamalpur	41	14	1	51	2.50	2032	8	2.05	26	0.020	17	0.05	52	0.000	56
Mymensingh	77	5	7	29	5.90	4363	8	1.63	33	0.018	22	0.15	46	0.002	45
Netrokona	63	9	14	14	2.32	2810	8	3.39	6	0.022	13	0.75	15	0.005	19
Sherpur	26	32	3	41	1.50	1364	8	2.16	23	0.019	20	0.25	40	0.002	40
Bogura	39	16	23	5	3.73	2920	8	1.31	43	0.013	37	0.77	14	0.008	9
Joypurhat	16	48	6	31	0.96	965	8	2.09	25	0.017	26	0.78	12	0.006	14
Naogaon	82	3	8	23	2.78	3436	8	3.68	5	0.024	11	0.36	32	0.002	37
Natore	27	30	9	21	1.86	1896	8	1.81	30	0.014	34	0.60	22	0.005	22
Chapai Nawabganj	65	7	19	7	1.84	1703	8	4.43	3	0.038	2	1.29	6	0.011	4
Pabna	53	12	7	29	2.91	2372	8	2.28	19	0.022	14	0.30	37	0.003	33
Rajshahi	67	6	15	12	2.92	2407	8	2.87	10	0.028	6	0.64	19	0.006	13
Sirajganj	64	8	15	12	3.36	2498	8	2.38	16	0.026	8	0.56	24	0.006	15
Dinajpur	56	11	30	2	3.32	3438	8	2.11	24	0.016	27	1.13	9	0.009	6
Gaibandha	53	12	12	16	2.56	2179	8	2.59	13	0.024	10	0.59	23	0.006	18
Kurigram	14	53	19	7	2.33	2296	8	0.75	56	0.006	55	1.02	11	0.008	8
Lalmonirhat	19	42	18	9	1.43	1241	8	1.66	31	0.015	32	1.58	4	0.015	3
Nilphamari	21	39	5	35	2.09	1580	8	1.25	47	0.013	38	0.30	38	0.003	30
Panchagarh	27	30	11	18	1.18	1405	8	2.86	11	0.019	18	1.17	8	0.008	10
Rangpur	37	19	17	11	3.17	2368	8	1.46	38	0.016	30	0.67	17	0.007	12
Thakurgaon	28	29	30	2	1.53	1810	8	2.28	18	0.015	31	2.44	1	0.017	1
Habiganj	111	2	13	15	2.36	2637	8	5.88	2	0.042	1	0.69	16	0.005	20
Moulvibazar	31	27	11	18	2.12	2799	8	1.82	28	0.011	43	0.65	18	0.004	28
Sunamganj	140	1	27	4	2.70	3670	8	6.49	1	0.038	3	1.25	7	0.007	11
Sylhet	61	10	11	18	3.86	3490	8	1.98	27	0.017	24	0.36	33	0.003	31
**Total**	**2142**		**538**		**165.16**										

Table 2 provides an overview of the overall lightning scenario in Bangladesh, comparing casualties and fatalities over the past eight years (2015–2022) across the nation's 64 districts spanning eight divisions. The district with the highest number of fatalities was Sunamganj, totaling 140, followed by Habiganj (111) and Naogaon (82). Conversely, districts with the lowest fatalities include Pirojpur (5), Bandarban (6), Gopalganj (6), Munshiganj (6), and Bagerhat (9). Sunamganj, Habiganj, and Naogaon are ranked highest for fatalities, while Satkhira (37), Dinajpur (30), and Thakurgaon (30) lead in injuries. Assessing fatality rates per million populations per year and density per square kilometer revealed Sunamganj (6.49), Habiganj (5.88), and Chapai Nawabganj (4.33) as the districts with the highest fatality rates, while Dhaka (0.13), Munshiganj (0.46), and Gazipur (0.50) had the lowest fatality rates. Moreover, fatality density per square kilometer was highest in Habiganj (0.042) and Chapai Nawabganj (0.038), with Sunamganj also at 0.038. Thakurgaon had the highest injury rate (2.44) and density (0.017). This study underscores that farmers and fishermen face the highest risk of lightning-related fatalities, particularly in the northeastern regions. Sunamganj District ranked first for fatalities, followed by Habiganj, while Satkhira topped the list for injuries, followed by Thakurgaon. Habiganj District exhibited the highest fatality density, while Chapai Nawabganj ranked second. Additionally, Pirojpur District was identified as the least affected by lightning fatalities in Bangladesh.

**Figure-3:**
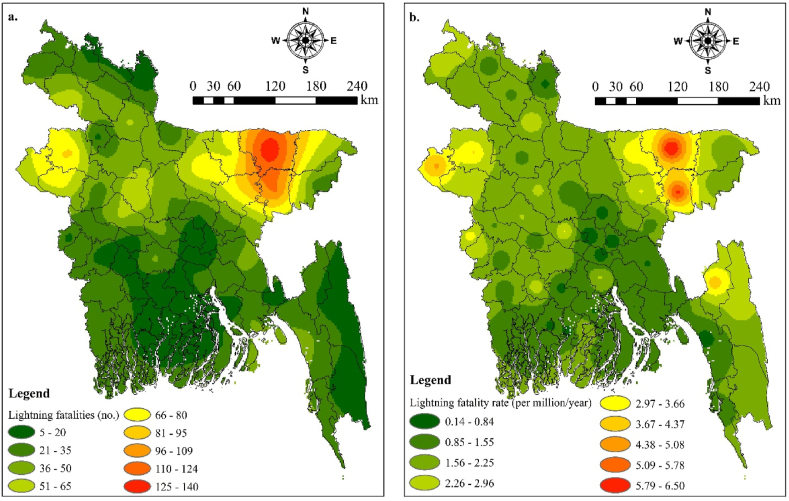
(a) Spatial distribution of lightning fatalities and (b) spatial distribution of lightning fatality rate of 64 districts in Bangladesh from the year 2015–2022.

Fig. 3a depicts the spatial distribution of total lightning fatalities across the 64 districts of Bangladesh from 2015 to 2022, while Fig. 3b illustrates the spatial variation of lightning fatality rates during the same study period. It is evident that the northeastern and northern regions of the country were disproportionately affected by lightning strikes, with the highest number of fatalities and fatality rates occurring in these areas. Particularly, Sylhet division emerged as the region most severely impacted by lightning strikes, with both the highest number of fatalities (exceeding 100 persons) (Fig. 3a) and fatality rates (exceeding 3.00 per million populations per year) (Fig. 3b). This vulnerability of the Sylhet division to lightning-related fatalities can be attributed to several factors. Firstly, the region experiences a high frequency of thunderstorm days, especially during the months from March to May, which significantly increases the incidence of lightning strikes [41]. Additionally, due to the substantial number of lightning-related deaths, the area is recognized as one of the most lightning-prone in the nation [9,15].

Moreover, the presence of a strong Sea Level Pressure (SLP) ridge extending from the northwest and a 500 hPa Geopotential Height (GpH) ridge crossing the northwest and extending to southeast Bangladesh facilitates severe lightning caused by thunderstorms in the region [9,42]. Furthermore, convective precipitation systems and lightning activity in Sylhet are influenced by the topographic forcing of the Chittagong hill tracts and the Shillong Plateau [15]. Furthermore, Sylhet's vulnerability is exacerbated by the presence of both a southerly to south-westerly low-level jet and a northerly to north-northwesterly subtropical jet stream, creating ideal conditions for thunderstorm and lightning formation [43]. This confluence of factors, confirmed by geostatistical models, suggests a higher risk of lightning disasters in the Surma Basin, which encompasses the Sylhet division. Thus, the geographic location of Sylhet within Bangladesh plays a significant role in its elevated susceptibility to lightning fatalities.

**Figure-4:**
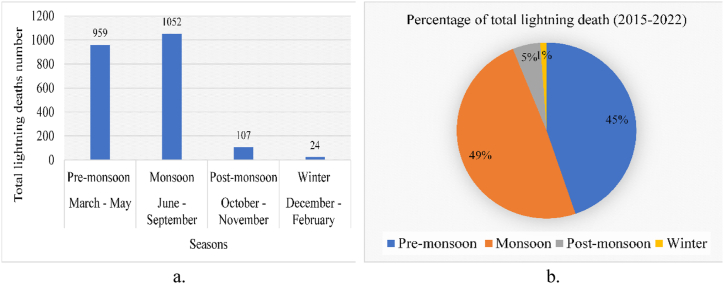
Lightning death toll (a) total death (2015–2020) (b) percentage of total lightning death (2015–2020) based on four different seasons.

The pre-monsoon and monsoon seasons exhibited the highest incidence of fatalities and injuries in Bangladesh, characteristic of its tropical monsoon climate (Fig. 4.). The pre-monsoon season spans from March to May and accounted for 959 lightning-related deaths, while the monsoon season, extending from June to September, recorded a total death toll of 1052. These two seasons collectively contribute to 94% of the total lightning fatalities from 2015 to 2022, with the monsoon season alone comprising 49% and the pre-monsoon season 45%. Conversely, the post-monsoon season (October to November) reported 107 deaths, constituting only 5% of the total, while the winter season (December to February) witnessed 24 deaths, amounting to just 1% of the total lightning fatalities. Clearly, the pre-monsoon and monsoon seasons stand out as the most vulnerable periods to lightning fatalities in Bangladesh, with significantly lower fatalities observed during the post-monsoon and winter seasons. Various factors contribute to this trend, including human activities and land conservation practices in specific areas. Lightning strikes are particularly frequent during the pre-monsoon (March to May) and monsoon seasons (June to September), coinciding with labor-intensive agricultural activities [44]. These seasons, characterized by heightened thunderstorm activity, pose the greatest risk for lightning-related incidents in Bangladesh.

In Bangladesh, the majority of the population relies on agriculture as a primary livelihood [45]. Consequently, a significant number of lightning-related deaths occur during the pre-monsoon and monsoon seasons due to the intensive agricultural activities conducted during these months, particularly in paddy fields [2]. Farmers are actively engaged in Boro harvesting, a crucial rice cultivation season that takes place during April–May, contributing to over 55% of the country's rice production [46]. The pre-monsoon and monsoon seasons are characterized by soaring temperatures and frequent lightning strikes, driven by intense solar radiation [47]. Furthermore, these seasons witness the highest density of cloud-to-ground lightning strokes [48], a significant contributing factor to lightning fatalities during this period. Conversely, the post-monsoon and winter seasons experience fewer lightning casualties in Bangladesh due to reduced thunderstorm activity and lower lightning stroke occurrences. Temperature variations also play a pivotal role in lightning activity, with surface temperature fluctuations influencing the seasonal and spatiotemporal distribution of lightning strikes. The rapid rise in temperatures during the pre-monsoon and monsoon seasons exacerbates lightning activity, whereas the relatively lower temperatures during the post-monsoon and winter seasons mitigate lightning incidences [49].

### 3.2 Seasonal variation and spatial distribution of thunderstorm days

**Figure-5:**
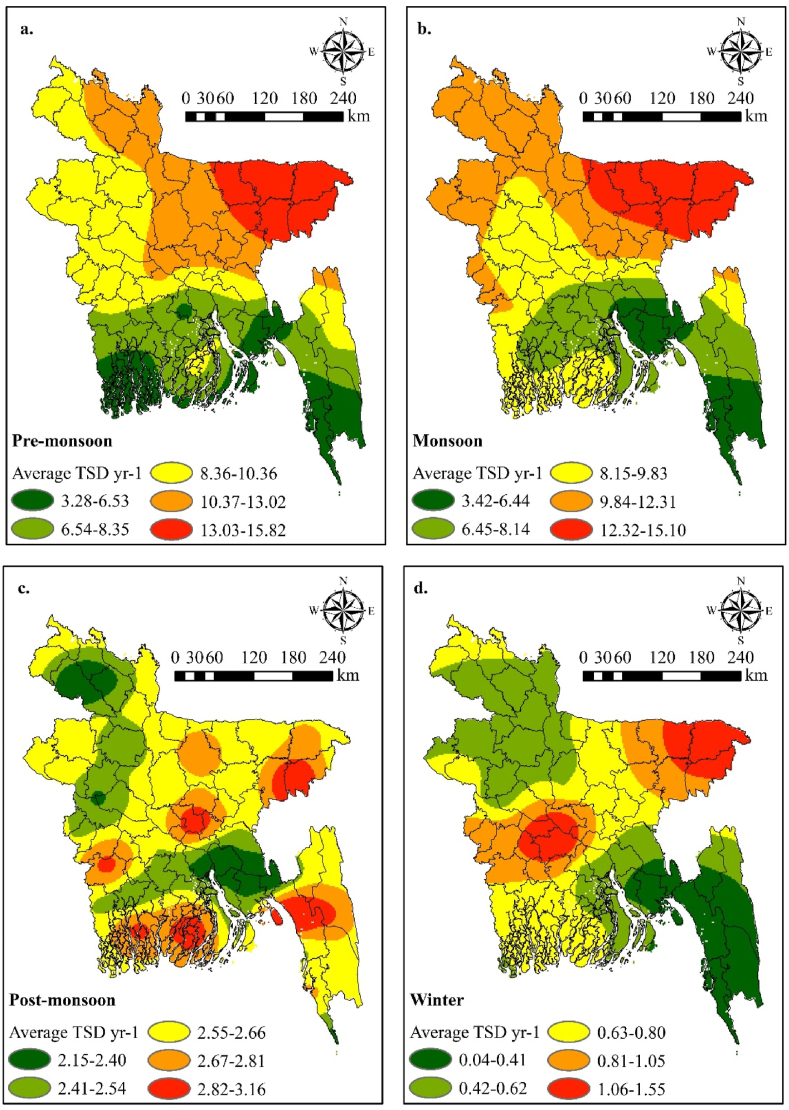
Seasonal spatial distribution of average thunderstorm days (TSD) (2015–2020): (a) pre-monsoon (March–May), (b) monsoon (June–September), (c) post-monsoon (October–November), and (d) winter season (December–February).

Due to its geographical positioning, Bangladesh encounters an elevated number of TSDs during the pre-monsoon and monsoon seasons, as depicted in Fig. 5. The data, spanning from 2015 to 2020 across 35 weather stations in the country, illustrates that both the pre-monsoon (Fig. 5a) and monsoon seasons (Fig. 5b) witnessed the highest frequency of TSDs, while the post-monsoon (Fig. 5c) and winter seasons (Fig. 5d) experienced comparatively fewer thunderstorm occurrences. Utilizing the IDW technique within GIS, TSDs maps have been generated based on station-specific data from the BMD. During the pre-monsoon and monsoon seasons, the northeastern, northern, and northwestern regions of Bangladesh exhibited moderate to maximum TSDs. Notably, Sylhet, situated in the northeastern region, registered the highest average TSDs (15.83 days) during the pre-monsoon season. The pre-monsoon season starts from March to May, whereas April and May are the warmest months in Bangladesh. Specifically, during the pre-monsoon season Srimangal (14.94 days), Mymensingh (12.28 days), Rangpur (11.83 days), Tangail (11.00 days), Faridpur (10.89 days), and Sayedpur (10.83 days) stations recorded the highest average TSDs, with Sylhet exhibiting the maximum (15.83 days). Similarly, during the monsoon season, Srimangal (14.88 days), Mymensingh (13.92 days), Rajshahi (12.38 days), and Jessore (11.33 days) stations reported the highest average TSDs, with Sylhet again leading (15.50 days). In contrast, the average TSDs were more prevalent in the southern and western regions during the post-monsoon season (Fig. 5c). Conversely, the winter season in Bangladesh witnessed very few TSDs, averaging less than two days. Overall, the northeastern, northern, and northwestern parts of Bangladesh experienced the highest average thunderstorm days, with the pre-monsoon and monsoon seasons contributing the most TSDs. This aligns with the fatality data (Table 1), which underscores that the northeastern districts, particularly within the Sylhet Division, endure the highest lightning-related death tolls.

### 3.3 Seasonal variation and spatial distribution of lightning flash frequency

**Figure-6:**
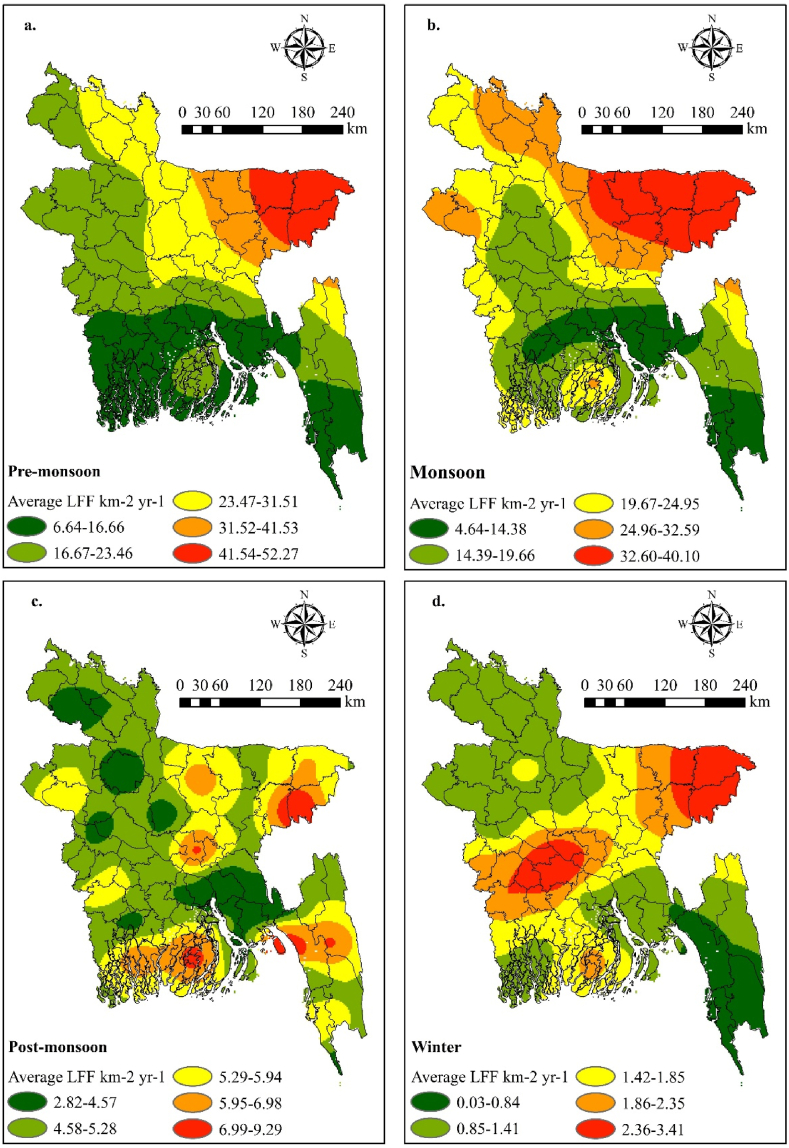
Seasonal spatial distribution of average lightning flash frequency (LFF) (2015–2020): (a) pre-monsoon (March–May), (b) monsoon (June–September), (c) post-monsoon (October–November), and (d) winter season (December–February).

The seasonal variation of LFF in Bangladesh from 2015 to 2020 is depicted in Fig. 6. The average LFF data has been compiled from 35 weather stations across the country. It is evident that the pre-monsoon and monsoon seasons experienced the highest LFF, with the pre-monsoon season exhibiting the maximum LFF in Bangladesh. During the pre-monsoon season, the LFF ranged from 41.54 to 52.27 $\text{fl}\;km^{-2}yr^{-1}$, while during the monsoon season, it ranged from 32.60 to 40.10 $\text{fl}\;km^{-2}yr^{-1}$. Conversely, the lowest LFF ranged from 6.64 to 16.66 $\text{fl}\;km^{-2}yr^{-1}$ in the pre-monsoon season and from 4.64 to 14.38 $\text{fl}\;km^{-2}yr^{-1}$ in the monsoon season. The second highest LFF range fell between 31.52 and 41.53 $\text{fl}\;km^{-2}yr^{-1}$ in the pre-monsoon season and 24.96 to 32.59 $\text{fl}\;km^{-2}yr^{-1}$ in the monsoon season (Fig. 6a & b). Furthermore, the southern part of Bangladesh exhibited the lowest LFF ranges during both the pre-monsoon and monsoon seasons. However, the highest and second-highest LFF ranges were observed during the pre-monsoon season, with the maximum affected area being during the monsoon season. The variation in LFF significantly influences lightning fatalities, with the highest occurrence of lightning-related deaths recorded during the pre-monsoon and monsoon seasons, as depicted in Fig. 4. Conversely, the post-monsoon season recorded lower lightning flash frequencies from 2015 to 2020. Specifically, during the pre-monsoon season, Srimangal (45.50 $\text{fl}\;km^{-2}yr^{-1}$), Mymensingh (30.67 $\text{fl}\;km^{-2}yr^{-1}$), and Rangpur (29.00 $\text{fl}\;km^{-2}yr^{-1}$) stations registered the highest LFF, with Sylhet experiencing the maximum (52.33 $\text{fl}\;km^{-2}yr^{-1}$). Similarly, during the monsoon season, Srimangal (38.42 $\text{fl}\;km^{-2}yr^{-1}$), Mymensing (36.38 $\text{fl}\;km^{-2}yr^{-1}$), Rajshahi (29.00 $\text{fl}\;km^{-2}yr^{-1}$), and Rangpur (28.13 $\text{fl}\;km^{-2}yr^{-1}$) stations reported the highest LFF, with Sylhet again leading (40.08 $\text{fl}\;km^{-2}yr^{-1}$). Fig. 6a and b highlight that the northeastern, northern, and northwestern regions of Bangladesh are characterized by the highest LFF, indicating that these areas are particularly prone to lightning strikes. Conversely, during the post-monsoon and winter seasons, the occurrence of lightning was significantly lower in the south and southwestern regions of the country. Moreover, districts within these regions such as Sunamganj (140), Habiganj (111), Naogaon (82), Kishoreganj (78), Mymensingh (77), Rajshahi (67), Chapai Nawabganj (65), Sirajganj (64), and Netrokona (63) experienced the highest number of lightning fatalities from 2015 to 2022, primarily due to the influence of LFF.

### 3.4 Reasons for the high LFF and TSDs in pre-monsoon and monsoon seasons

Bangladesh stands out as one of the most lightning-prone nations globally, owing to its unique geographic positioning. This study revealed a stark disparity in thunderstorm and lightning occurrences between Bangladesh's pre-monsoon and monsoon seasons compared to the other periods. Notably, the pre-monsoon season, spanning from March to May, and the subsequent monsoon season exhibited a significantly heightened frequency of thunderstorm days and lightning flash events. Various atmospheric factors influence the likelihood of lightning strikes, including temperature differentials, air pressure gradients, and moisture dynamics. Moreover, Bangladesh experiences a substantial temperature shift between winter and the pre-monsoon months, with a marked upsurge in temperatures observed in March, indicating pre-monsoon atmospheric instability [48]. The mean summer temperature range in Bangladesh falls between 27.8 °C and 29 °C, while the average winter temperature range spans from 18.5 °C to 21 °C. The winter season concludes in February, followed by a notable rise in temperature during the pre-monsoon period from March to May, with April emerging as the warmest month in Bangladesh. During the pre-monsoon period, Bangladesh's climate exhibits distinct features such as a moisture gradient or dry line, vertical wind shear, varied land surface topography, subsidence of the Hadley circulation, and low-level convergence. These atmospheric conditions create an environment conducive to heightened severe storm occurrence and lightning activity [49]. Lightning in the pre-monsoon and monsoon seasons primarily arises from the atmospheric instability within cloud formations, driving the formation of thunderstorms and subsequent lightning strikes.

An influential factor contributing to convective activities over Bangladesh is the Gangetic plains of India, which extend into Bangladesh, particularly through the nation's central regions. The presence of a robust Sea Level Pressure (SLP) ridge and a 500 hPa geopotential height (GpH) ridge spanning northwest to southeast Bangladesh further enhances the likelihood of thunderstorm-mediated severe lightning events. During the pre-monsoon season, lightning activity in Bangladesh exhibits a shift from northeastern to southwestern regions, correlating with the movement of the most robust 500 hPa GPH anomaly center from May to June [50]. The northeastern and northwestern regions of Bangladesh consistently experience the highest frequency of thunderstorm days and lightning flashes. Specifically, the period from mid-April to late May witnesses a peak in lightning strikes, with the pre-monsoon and monsoon seasons exhibiting twice as many morning strokes compared to afternoon strokes, a phenomenon uncommon in the broader Indian subcontinent [51]. The pronounced temperature anomaly and associated convective precipitation systems, driven by topographic forces from the Shillong Plateau and the Chittagong Hill Tracts, contribute significantly to heightened pre-monsoon lightning activity. Additionally, during the pre-monsoon season, a low-level jet stream moving from south to southwest facilitates the transportation of moisture from the Bay of Bengal, further fueling thunderstorm development in Bangladesh.

Furthermore, the presence of the north-to-northwesterly subtropical jet stream further enhances conditions conducive to frequent pre-monsoon lightning activity [50]. Conversely, during the post-monsoon and winter seasons, temperatures are comparatively lower than in the preceding periods. Winds predominantly blow from the northeast, carrying dry air towards the sea. This wind carries less moisture in the atmosphere as it traverses over the country. Consequently, the scarcity of moisture-laden air masses contributes to fewer thunderstorms and lightning occurrences during the winter season in Bangladesh.

## 4 ISS LIS NRT lightning flash events over Bangladesh

According to data from the ISS, between 2017 and 2022, a total of 667,136 lightning flash events were detected in Bangladesh by the LIS under the NRT mission. The majority of these lightning occurrences were observed during the pre-monsoon season, accounting for 56.93% of the total, while 33.59% were recorded during the monsoon season. Comparatively, the post-monsoon season (2.54%) and the winter season (6.94%) witnessed significantly fewer lightning flash events (refer to Table 3).

**Table-3:** Seasonal pattern of lightning flash events over Bangladesh (2017–2022).

Seasons	Months	Flash events	Seasonal total	%	Seasonal (%)
Pre-monsoon	March	51602	379816	7.735	56.93
	April	115076		17.249	
	May	213138		31.948	
Monsoon	June	94256	224086	14.128	33.59
	July	24529		3.677	
	August	42601		6.386	
	September	62700		9.398	
Post-monsoon	October	16940	16945	2.539	2.54
	November	5		0.001	
Winter	December	4984	46289	0.747	6.94
	January	11		0.002	
	February	41294		6.190	
**Total**		**667136**		**100.000**	**100.00**

**Figure-7:**
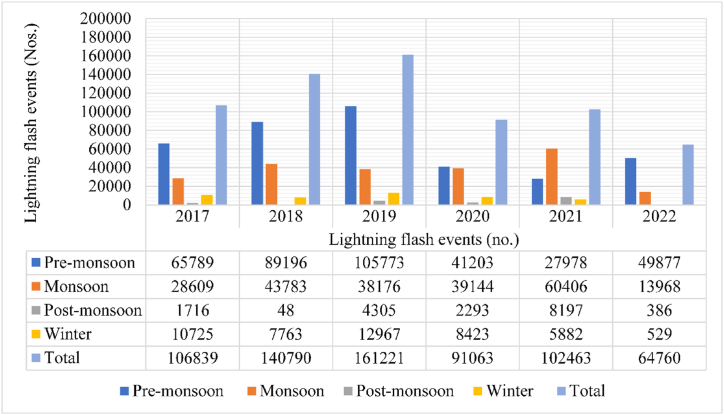
Temporal change of lightning flash events over Bangladesh.

The analysis revealed a concentration of lightning flash events during the pre-monsoon period, particularly from April to June, with May exhibiting the highest frequency, representing 31.95% of all events during the study period. Following May, April (17.24%), June (14.12%), and September (9.39%) also experienced substantial lightning activity (refer to Table 3). Conversely, November and January recorded the lowest occurrences of lightning flash events. The temporal distribution of annual lightning flash events is illustrated in Fig. 6, depicting fluctuations over the years. Notably, the highest count of lightning events occurred in 2019, with 161,221 flashes recorded, while the lowest count was observed in 2022, totaling 64,760 flashes. In 2018, the second-highest number of flash events was recorded, totaling 140,790 (see Fig. 7).

Bangladesh's geographical positioning significantly influences its susceptibility to weather and climate-related disasters, particularly lightning-related incidents. Situated in South Asia with a tropical climate marked by distinct monsoon seasons, Bangladesh experiences a disproportionate number of lightning-related fatalities, primarily concentrated in its northeast and northwest regions. These areas are characterized by flat terrain and expansive agricultural landscapes, making them particularly susceptible to lightning strikes. The proximity of Bangladesh's northern region to the Shillong Plateau and the foothills of the Himalayas further exacerbates the prevalence of lightning events. Warm and moisture-laden air from the Bay of Bengal envelops the country, creating conditions ripe for lightning formation. The process of air forced to ascend over rugged terrain triggers cooling and condensation, fostering the development of thunderstorms and subsequent lightning discharges. This study underscores the vulnerability of Bangladesh's northeastern region, accentuated by the presence of Haor, a distinctive wetland ecosystem unique to this area. Haor's presence exacerbates the region's susceptibility to lightning strikes, amplifying the risks faced by farmers working in the surrounding fields during cultivation seasons. The combination of geographical features and climatic conditions in Bangladesh heightens the likelihood of lightning-related incidents, particularly in areas with low-lying plains and wetlands like Haor.

This study highlights the heightened vulnerability of the Sylhet Division, located in the northeastern part of Bangladesh, to lightning occurrences and associated damages. This assertion finds support in various research works, including those by Holle et al. (2019) [9], Dewan et al. (2017) [13], Islam & Schmidlin (2020) [22], Rahman et al. (2023) [41], Mazumder et al. (2021) [52], and Rahman et al. (2022) [53]. Furthermore, the study identifies agricultural activities, such as crop farming and fishing, as the primary contexts in which most lightning fatalities occur. This finding aligns with the research conducted by Holle et al. (2019) [9]. While the monsoon period emerges as the timeframe with the highest incidence of lightning fatalities in Bangladesh, there remains a notable gap in concrete research regarding the role of climate change in exacerbating these fatalities. However, the changing patterns of the monsoon, attributed to global warming and climate change, suggest a potential link to the observed increase in lightning-related risks. Kabir & Jakariya (2021) [43] and Mazumder et al. (2021) [52] have explored this connection, indicating a compelling relationship between climate change and lightning risk in Bangladesh. Given the significance of Bangladesh in this context, further research is warranted to elucidate the intricate relationship between lightning damage and climate change, providing valuable insights for mitigation and adaptation strategies in the face of evolving environmental conditions.

## 5 Conclusion

It is evident that Bangladesh is facing an alarming increase in lightning casualties, attributed to drastic shifts in weather patterns. As a consequence of its geographical location, Bangladesh ranks among the countries most prone to lightning fatalities globally. This study utilized ArcGIS to analyze spatial distribution data derived from local BMD records and LIS NRT data from the ISS to assess lightning-related incidents from 2015 to 2022. The analysis revealed a concentration of lightning fatalities and events during the pre-monsoon and monsoon seasons, with the northern and northeastern regions of Bangladesh emerging as the most vulnerable areas to lightning disasters. Conversely, the southern parts of the country exhibited lower susceptibility to such incidents. Notably, districts like Sunamganj, Habiganj, and Naogaon reported the highest number of lightning fatalities, with Sunamganj registering the highest fatality rate at 6.49, followed by Habiganj at 5.88, and Chapai Nawabganj at 4.33 per million population.

These findings align with previous studies conducted in Bangladesh. Additionally, this study introduced NASA instrument data, integrating BMD and ISS LIS lightning datasets, to provide a comprehensive understanding of the lightning scenario in Bangladesh. Despite the surge in lightning fatalities, addressing this issue remains challenging due to socio-economic constraints and technological limitations. The effectiveness of technology in mitigating lightning risks across vast territories remains uncertain, prompting the need for a comprehensive plan and guidelines. Further research efforts should focus on developing robust policies to effectively combat this pressing issue and safeguard the population from lightning-related hazards.

## Limitation

This study relies on secondary data sources from various research papers and government institutions. While data on thunderstorm days and lightning flash frequency was readily available from the BMD for the period 2015–2020, limitations arose in acquiring data on lightning flash events. We were able to access data from the ISS' LIS under the NRT mission, but only for the period 2017–2022. This discrepancy in data availability across different sources created some challenges in analysis. Additionally, this study primarily focuses on data analysis and does not delve into proposing comprehensive plans or policy recommendations to address the current lightning fatality issue. Further research and stakeholder engagement would be necessary to develop effective mitigation strategies.

This research received no external funding.

## Data availability statement

The data used in this study will be made available upon request.

## CRediT authorship contribution statement

**Ferdous Ahmed:** Writing – review & editing, Writing – original draft, Visualization, Validation, Supervision, Resources, Project administration, Investigation, Formal analysis, Data curation, Conceptualization. **Sakib Hasan:** Writing – review & editing, Writing – original draft, Software, Resources, Methodology, Investigation, Formal analysis, Data curation. **I.M. Mahbubul:** Writing – review & editing, Jayatra Mandal, Methodology, Data curation. **Muhammad Abul Kalam Mallik:** Supervision, Data curation. **M. Nafiz Hossen:** Formal analysis, Data curation.

## Declaration of competing interest

There is no conflict of interest among authors.

## Footnotes

1 A Haor is a bowl-shaped, shallow wetland ecosystem in northeastern Bangladesh, also known as a backswamp. During the monsoon season, Haors collect surface runoff water from rivers and canals, transforming into vast stretches of turbulent water.
